# What Is Needed to Move Toward Single-Step Diagnosis of Current HCV Infection?

**DOI:** 10.1093/infdis/jiad453

**Published:** 2023-10-13

**Authors:** Jordan J Feld

**Affiliations:** Toronto Centre for Liver Disease, Toronto General Hospital, University Health Network, University of Toronto, Toronto, Ontario, Canada

**Keywords:** cascade of care, HCV core antigen, HCV elimination, point-of-care (POC) RNA, reflex testing

## Abstract

Despite remarkable therapeutic advances, hepatitis C virus (HCV) infection continues to be a major global problem. While the development of highly effective direct-acting antivirals has ensured that almost all those who are treated achieve viral cure, progress toward HCV elimination globally has stalled due to challenges upstream of treatment in the cascade of care, namely diagnosis and linkage to care. The major challenge continues to be the relative complexity of HCV diagnosis with the current requirement for a confirmatory HCV RNA test after an initial antibody-positive result. In this review, challenges with the current paradigm are highlighted with a focus on new technologies, as well as simple strategies using existing tools, which may simplify diagnosis and improve linkage to care and treatment. To achieve HCV elimination, improvements in the HCV diagnostics field to allow for a simple single-step diagnosis are required.

Despite the remarkable progress in therapeutics for hepatitis C virus (HCV) infection with the development of direct-acting antivirals (DAAs) that cure over 95% of those treated, few countries globally are on track to meet the ambitious targets of the World Health Organization to eliminate HCV as a public health threat by the year 2030 [1]. DAAs have ensured that almost all who initiate treatment are cured, but the challenges to achieving elimination lie further upstream in the cascade of care with initial diagnosis and subsequent linkage to care and treatment.

Diagnosis of HCV infection remains relatively complex due to the multistep process involved. Current paradigms require an initial test for HCV antibodies that confirms exposure to the virus but does not indicate whether infection is current, followed by a second test to confirm active infection. Most often, the follow-up assay tests for HCV RNA in the blood as an indicator of current, ongoing infection. For individuals 'accessing care, this process can be challenging.

Each test requires a visit to a provider, and unless reflex testing for HCV RNA is performed on the original blood sample, a follow-up at a laboratory to have more blood taken, a subsequent visit to receive results, which may take many days or even weeks to arrive, and only then can a discussion about HCV treatment begin. Many people get lost to follow-up along the way; particularly when HCV is not a major priority due to its relative lack of symptoms and slow progression [2]. Additionally, barriers such as poor venous access due to past injection drug use as well as competing priorities that are more time-sensitive in the marginalized populations that are disproportionately affected by HCV, may compound loss to follow-up [2]. This multistep process is also particularly problematic in rural or remote regions and in low- and middle-income countries (LMIC) where access to providers and laboratories may be limited, further lengthening timelines [3].

In this review, considerations of what would be required to simplify this process to allow for a reliable diagnosis of HCV infection with a single step are explored. While there is not a current solution to this problem, there are some promising developments that may make a single-step diagnosis feasible, a key step toward advancing HCV elimination efforts.

## THE PREFERRED PARADIGM

Ideally, a rapid, point-of-care (POC) test for HCV viremia could be used as a screening test to identify those with active infection who require treatment. If the additional pretreatment work-up (eg, hepatitis B virus [HBV], human immunodeficiency virus [HIV], basic laboratory testing including a fibrosis assessment) could also be done using POC devices, treatment could be initiated immediately, which would greatly reduce loss to follow-up, particularly in marginalized populations.

Currently, there are no true POC assays for HCV RNA and although HIV POC tests exist, those for HBV are not approved in many regions. Because HBV testing is required before starting HCV therapy, approval of POC hepatitis B surface antigen (HBsAg) tests along with novel HCV assays should be a priority to ensure that novel HCV paradigms actually accelerate treatment initiation. Access to either POC blood tests or to transient elastography for fibrosis assessment is extremely limited, which would be another requirement to enable immediate treatment starts following HCV diagnosis. It is important to consider these other requirements along with improving HCV diagnostics to ensure advances lead to expected acceleration of treatment.

## REFLEX HCV RNA TESTING

The implementation of reflex HCV RNA testing, in which a test for HCV RNA is automatically performed when a test comes back positive for HCV antibody, has been an important advance. In regions without reflex testing, 20% to as high as 50% of people who test HCV antibody positive do not have a follow-up HCV RNA performed, constituting one of the major drop-offs in the HCV cascade of care. When Quest diagnostics, a large commercial laboratory in the United States, adopted reflex HCV RNA testing, the rate of follow-up HCV RNA testing within 30 days of a positive HCV antibody test went from 40% to 95%. Similarly, Spanish data showed that the rate of RNA testing increased from 52% to 91% with introduction of reflex testing [4]. Even with reflex testing, challenges with inadequate sample volume or sample mishandling, prevent laboratories from getting to universal HCV RNA testing for all antibody-positive samples.

Although reflex testing is an important step and should be standard practice globally, implementation is limited in many regions of the world, including in many high-income countries, particularly in rural and remote communities. Although laboratories have cited challenges to move samples from the serology section of a laboratory to the site of nucleic acid testing, particularly if these tests are not done in the same place, this should be weighed against the inconvenience and cost to a patient to return to a provider and provide a second sample for follow-up testing. Some laboratories require collection of a second sample at the time of initial antibody testing, which improves the reliability of the reflex test, but greatly increases the cost of HCV screening because most screened individuals test HCV negative. Even where reflex testing has been adopted, turnaround times for HCV antibody and RNA results are often prolonged, particularly if testing volumes are low, leading laboratories to wait for enough samples for batch testing. Reflex HCV RNA testing should already be standard practice globally and efforts to ensure its universal adoption should be a priority.

## IS THERE ANY VALUE IN HCV ANTIBODY TESTING?

The primary purpose of HCV screening is to identify those with current infections who require treatment. One potential downside to moving to a single test for current HCV infection would be missing those who test HCV-antibody positive but HCV RNA-negative (Ab^+^/RNA^−^), indicating past exposure to HCV with spontaneous viral clearance. Studies of people who spontaneously cleared HCV have shown that after controlling for acquisition risks, particularly injection drug use, the long-term survival of those with resolved HCV infection is comparable to the non-HCV–exposed general population [5]. Therefore, there is no clear medical reason to know if someone is HCV antibody-positive and the result may create confusion for providers and may cause anxiety and possibly be stigmatizing, including with health and life insurers, for patients. However, past HCV exposure is likely an indication of past risk activities. Although reliable screening for past and current drug use and other potential HCV exposures should be part of comprehensive medical assessments, it is likely that many risk activities remain unrecognized. The positive antibody test may facilitate a deeper exploration of past risk activities, which may indicate other health risks, as well as the possibility of recurrent risk exposures (eg, relapse to drug use); however, whether this trigger to identify risk factors is worth the cost and potential consequences is questionable. The current antibody followed by RNA testing approach may also, at least marginally, improve sensitivity and specificity for HCV diagnosis. During acute HCV infection, people may test transiently HCV RNA negative. A newly positive antibody result would prompt repeat HCV RNA testing that may not be recognized if the antibody result were not available. Although less common with current assays and laboratory practices, false-positive RNA results may also rarely be an issue. However, at a conference in 2021 by the Centers for Disease Control and Prevention (CDC) and the Association of Public Health Laboratories (APHL) on the topic of HCV diagnostics, the majority of stakeholders felt that the value of antibody testing was limited, and the focus should be to move to a single test for active HCV infection.

## OPTIONS FOR ONE-STEP DIAGNOSIS OF HCV INFECTION

Diagnosis of an active HCV infection requires confirmation of viremia. Although HCV RNA testing is the most commonly used strategy to assess for viremia, any assay that detects a portion of the virus in the blood could also be used. Unlike many other viral infections, the HCV viral load is not an important determinant of disease progression or response to treatment. Accordingly, tests for viremia can be qualitative rather than quantitative, provided that they have adequate sensitivity to detect low levels of circulating virus.

### HCV RNA

HCV RNA testing is available and could in theory already be used as a single-step tool for HCV diagnosis. The limitations of HCV RNA testing are cost and turnaround time. Except in populations with extremely high HCV prevalence (eg, people who inject drugs), the vast majority of HCV testing in any population screening effort will be negative. The rationale to start with HCV antibody is that it is an inexpensive test that can be done in large numbers of people to reduce the amount of HCV RNA testing that is required. It is unlikely that HCV RNA testing using current technologies could be made cheap enough to be a cost-effective strategy for population screening.

HCV RNA is most commonly done in a central laboratory using high-throughput real-time polymerase chain reaction (PCR). High-volume testing facilities will run tests frequently but in lower-volume settings, samples will be collected until adequate numbers for a batch have been received. This approach is cost-efficient for the laboratory, but can significantly delay result reporting, particularly when added to transport times if samples are traveling long distances to a central laboratory. These delays and the potential resultant loss to follow-up should be factored into the apparent economic benefits of batch testing.

Access to true POC HCV RNA would be a major advance. The GenXpert system allows for what is sometimes referred to as “near-care” testing, which is still a major improvement over sending samples to a central laboratory. The GenXpert platform is available in many LMICs for tuberculosis, HIV, and other testing panels. HCV RNA can be measured from serum or plasma with very high reliability and analytical performance equivalent to commercial laboratory-based assays. The test was adapted to be used on finger-prick whole blood, avoiding the need for phlebotomy and sample processing, with no reduction in sensitivity or specificity [6, 7]. The platform has been tested in clinical trials and real-world settings with very good performance and is now approved in Europe and many LMICs. Turnaround time is still a limitation that keeps this test from being truly POC. The time to positivity depends on viral load, with samples above 6 log IU/mL testing positive in approximately 30 minutes compared to those with viral loads <3 log IU/mL, which take approximately 60 minutes. In a study in the ETHOS cohort in Australia, over 80% of positive tests were completed in <40 minutes, but confidently calling a test negative required 57 minutes [8]. However, a recent meta-analysis found that even with current limitations, POC HCV RNA testing improved the cascade of HCV care by decreasing time from antibody to RNA testing but more importantly, by improving treatment uptake, particularly when integrated into a simplified care model [3].

The GenXpert has been evaluated as a single-test approach to HCV diagnosis. In studies in supervised injection sites (SIS), first in Canada [9] and subsequently in Australia [10], the tests were offered to clients of the SIS. In Toronto, the prevalence of HCV viremia was 42% and in Melbourne, it was 28%. Both studies reported high acceptability by both staff and clients with very good linkage to care for those who tested positive. It is likely that with such high prevalence, and likely much higher antibody prevalence, that going directly to HCV RNA testing is a cost-effective strategy; however, there are few settings with such high prevalence and the specific threshold at which initial RNA testing would be the most efficient approach may vary by setting and pricing. The SIS is also relatively unique in that clients stay for a long time, making turnaround time less of a concern. Other issues with the GenXpert are the cost of the device, which is inaccessible for many community testing sites, and some challenges with test failure rate that improves with experience [6]. Other similar platforms are in development.

### HCV Core Antigen

An alternative to HCV RNA measurement is the use of HCV core antigen (Ag). As a direct measure of a structural viral protein, detection of core Ag in the blood is equivalent to detecting HCV RNA. HCV core Ag is highly specific and correlates well with HCV RNA but is less sensitive, with existing assays reporting an analytical sensitivity equivalent to approximately 3000 IU/mL of HCV RNA [11]. Current core Ag platforms are automated but require a central laboratory and with current technology, POC assays have proven difficult to develop due to challenges with virion lysis, dissociation of the core Ag from antibody complexes, and the need for signal amplification to improve sensitivity. However, core Ag tests can be done on the same sample as HCV antibody tests.

Although core Ag test is cheaper than HCV RNA testing and could theoretically be used as a first-line test, the sensitivity may be a relevant issue. In a large study of 62 000 samples from patients with chronic HCV infection of varying genotypes, Freiman and colleagues reported that 97% of samples were above 1000 IU/mL and 95% were above 3000 IU/mL[12]. Current commercial HCV RNA assays have sensitivity of 12–15 IU/mL and the GenXpert POC RNA test has a lower limit of detection of 40 IU/mL. Notably, Freiman et al found that the factors associated with an HCV RNA level below 1000 IU/mL were genotype 3 infection, HIV coinfection, and the presence of cirrhosis, populations that would be particularly concerning to miss [12]. In large real-world studies of core Ag testing, sensitivity ranges from 87% to 94% compared to HCV RNA, with most core Ag-negative samples showing low HCV RNA levels [13, 14]. Mutations in the core region that interfere with detection may also give false-negative results [15]. Despite this observation, the European Association for the Study of the Liver (EASL) has recommended that new assays for HCV viremia must only be able to detect samples equivalent to HCV RNA levels of 1000 IU/mL or greater [16].

If testing in a low-prevalence population (eg, 1%–2%), even with 97% sensitivity, the negative predictive value of a test would still be extremely high approximately 99.9%. However, in higher-prevalence populations (eg, PWID with prevalence approximately 40%), the negative predictive value would only be 98%. At its current sensitivity, core Ag test would likely have to be used with a paradigm of testing all core Ag-negative/HCV antibody-positive tests for HCV RNA [13]. The cost-effectiveness of this strategy would likely depend on the viremic prevalence among antibody-positive people, which will go down as more and more people are treated. Ultimately, the availability of HCV core Ag test could be helpful if algorithms could be designed to test core Ag, with a plan to treat if positive but follow-up with reflex antibody and then RNA testing in those who test core Ag negative/HCV antibody positive. The cost-effectiveness and efficiency of such a strategy would have to be assessed to determine in which settings it would be most useful. Development of a POC core Ag test, ideally with improved sensitivity over existing assays, would be a major advance and would likely be not only cost-effective but significantly cost-saving [17] (Table 1).

**Table 1.** Examples of Pros and Cons of Current Testing Modalities

Testing Modality	Advantages	Disadvantages
Ab followed by reflex HCV RNA	Cost efficient Accurate	Loss to follow-up Burden on patient (time/anxiety)
Ab followed by core Ag	Cost efficient	Low sensitivity
POC Ab followed by POC HCV RNA	Rapid	Relatively costly Turnaround time
Immediate POC HCV RNA	Rapid	Costly except with very high prevalence
POC HCV Core Ag	Rapid Cost efficient	Not available—technically difficult

Abbreviations: Ab, antibody; Ag, antigen; HCV, hepatitis C virus; POC, point of care.

## MATCHING THE TEST TO THE SETTING

Although it would be preferable to have a single test for HCV diagnosis in all settings, currently that is difficult to achieve in a cost-effective manner in most scenarios. However, thoughtful considerations to match the testing strategy to the setting, may allow for use of more expensive strategies in high-yield settings if savings can be leveraged from use of lower-cost approaches where they are effective.

In addition to the testing modality itself, it is important to consider the population being tested in terms of likelihood of follow-up and expected HCV prevalence, the sample type (eg, finger-prick vs phlebotomy), and the geography in terms of access to providers and laboratory services. The current strategy of sequential antibody followed by HCV RNA testing, even without reflex testing, works well in populations who are seen reliably such as birth cohort screening or testing in opiate agonist therapy clinics where clients come back frequently [18, 19]. In contrast, POC testing is clearly required in certain settings such as screening drives, outreach work to marginalized populations, and in prison/jail [20]. In all of these settings, a POC HCV antibody could be performed from a fingerstick without the need for phlebotomy with a plan for immediate HCV RNA testing in those who test antibody positive. HCV RNA could be tested on a platform like the GenXpert or alternatively could be collected on a dried blood spot (DBS) card and sent to the laboratory for testing, provided strategies were in place to ensure follow-up and linkage to care. DBS collection is particularly useful in rural and remote regions with limited providers, where samples can be collected by peers, or even by self-collection, ensuring results are ready when healthcare providers come to the community [3, 21]. In very-high-prevalence settings, particularly if follow-up may be uncertain, like SIS, initial testing with HCV RNA, ideally from a system like GenXpert that does not require phlebotomy, would be preferred [9] (Table 2). Significant coordination at a systems level would be required to enable first-line HCV RNA testing where it is most needed by reducing costs in other settings.

**Table 2.** Matching the Testing Paradigm to the Setting

Characteristic	Example Populations	Preferred Paradigm
Reliable follow-up	Birth cohort stable OAT	1. Standard 2-step Ab followed by HCV RNA
Short interaction with uncertain follow-up	Jail PWID Screening drive	1. POC Ab → POC RNA 2. POC Ab → DBS collection
Very high prevalence	SIS Active PWID Prison (?)	1. POC HCV RNA/(Core Ag)
Limited healthcare providers	Rural/remote Self-testing	1. DBS → Ab and HCV RNA

Abbreviations: Ab, antibody; Ag, antigen; DBS, dried blood spot; HCV, hepatitis C virus; OAT, opiate agonist therapy; POC, point of care; PWID, people who inject drugs; SIS, supervised injection site.

Strategic use of tests may also be helpful. For example, Smookler and colleagues reported that using the OraQuick POC HCV antibody test, the antibody became positive much faster in those with current viremia than in people who had cleared HCV either with treatment or spontaneously [22]. They narrowed this down and showed that in 227 viremic patients, none tested positive beyond 5 minutes despite the label-recommended waiting time of 20–40 minutes before reading the result. With this 5-minute rule, any test that is not positive in 5 minutes does not require HCV RNA testing, which reduces overall HCV RNA testing and also allows higher throughput during screening initiatives [22]. The shorter wait time also translates to less loss to follow-up with one study reporting 18% of people did not wait 20 minutes to get the OraQuick result where 99.4% waited the 5 minutes required with this approach [23].

Modeling studies can help determine the best testing strategy for each location. Adee and colleagues developed the Hep C Testing Calculator to evaluate different testing and care pathways in Georgia [24]. Although they focused on 2-step diagnostic pathways, the approach is useful and could be modified to evaluate determinants (eg, viremic prevalence, price, turnaround time) of cost-effectiveness for 1-step paradigms to ensure the optimal approach is used for each setting.

## FUTURE APPROACHES

The COVID-19 pandemic has reshaped thinking about diagnostics. Firstly, tools for severe acute respiratory syndrome coronavirus 2 (SARS-CoV-2) detection were developed extremely rapidly using multiple platforms. In addition to standard PCR from nasal swabs, antigen tests were quickly developed that are inexpensive, relatively sensitive and specific, and could be used by individuals for self-testing [25]. The explosion of diagnostics during the pandemic is a testament to what can be achieved quickly when required and when funding is available to support innovation. To date the diagnostics industry in HCV has not kept pace with therapeutic developments in the field.

Hopefully, some of the technologies that were pioneered for COVID-19 can be repurposed or modified for other pathogens, including HCV. POC antigen tests would be extremely helpful, and rapid nucleic acid testing with standard PCR platforms (eg, Abbott ID NOW) could also be used for HCV. Novel approaches such as using CRISPR-Cas systems for highly specific diagnostics with the potential for low-cost implementation were also advanced and could be applied to HCV and other pathogens [26]. These approaches could enable broader adoption of self-testing, which may help reach certain populations who are currently not accessing care.

The recent decision of the US Food and Drug Administration (FDA) to reclassify nucleic acid-based HCV RNA tests from class III to class II in December 2021, will hopefully facilitate development of new testing platforms. Class III devices require premarket approval from the FDA, which is a long and expensive process. In contrast, class II devices require general and specific controls and in the case of HCV RNA, premarket notification to the FDA; however, this overall process is much faster, which will hopefully allow smaller companies with innovative strategies to enter the market. Integration of improved diagnostics into national and global HCV elimination plans, including funding initiatives, will also need to be a priority.

## CONCLUSION

To accelerate HCV elimination efforts, diagnostic paradigms will have to be simplified. Development of single-step approaches to diagnosis of current HCV infection is likely possible but current tools have significant limitations including cost, turnaround time and sensitivity. The rapid development of high-quality diagnostics for SARS-CoV-2 will hopefully lead to innovation in other fields. Until simple POC tests for viremia are available, it will be important to optimally match testing approaches to the population and the setting to ensure that testing tools are most efficiently used. A new regulatory environment in the United States and guidelines with less-stringent requirements will hopefully encourage entry into the HCV diagnostics field and accelerate the path to a single-step HCV diagnosis.

## Notes

***Financial support.*** No financial support was received for this work.

***Supplement sponsorship.*** This article appears as part of the supplement “Towards a Single-Step Diagnosis of Hepatitis C Virus Infection,” sponsored by the Centers for Disease Control and Prevention.

## References

[1] Polaris Observatory HCV Collaborators . Global change in hepatitis C virus prevalence and cascade of care between 2015 and 2020: a modelling study. Lancet Gastroenterol Hepatol 2022; 7:396–415. 35180382 10.1016/S2468-1253(21)00472-6

[2] Grebely J , Applegate TL, Cunningham P, Feld JJ. Hepatitis C point-of-care diagnostics: in search of a single visit diagnosis. Exp Rev Mol Diagn 2017; 17:1109–15. 10.1080/14737159.2017.1400385 29088981

[3] Smookler D , Beck A, Head B, et al A collaborative approach to hepatitis C testing in two first nations communities of northwest Ontario. Can Liver J 2022; 5:329–38. 36133895 10.3138/canlivj-2021-0031 PMC9473560

[4] López-Martínez R , Arias-García A, Rodríguez-Algarra F, et al Significant improvement in diagnosis of hepatitis C virus infection by a one-step strategy in a central laboratory: an optimal tool for hepatitis C elimination? J Clin Microbiol 2019; 58:e01815–19. 31694971 10.1128/JCM.01815-19 PMC6935937

[5] Omland LH , Christensen PB, Krarup H, et al Mortality among patients with cleared hepatitis C virus infection compared to the general population: a Danish nationwide cohort study. PLoS One 2011; 6:e22476. 21789259 10.1371/journal.pone.0022476 PMC3138785

[6] Catlett B , Hajarizadeh B, Cunningham E, et al Diagnostic accuracy of assays using point-of-care testing or dried blood spot samples for the determination of hepatitis C virus RNA: a systematic review. J Infect Dis 2022; 226:1005–21. 35150578 10.1093/infdis/jiac049

[7] Grebely J , Lamoury FMJ, Hajarizadeh B, et al Evaluation of the Xpert HCV viral load point-of-care assay from venepuncture-collected and finger-stick capillary whole-blood samples: a cohort study. Lancet Gastroenterol Hepatol 2017; 2:514–20. 28442271 10.1016/S2468-1253(17)30075-4

[8] Grebely J , Catlett B, Jayasinghe I, et al Time to detection of hepatitis C virus infection with the Xpert HCV viral load fingerstick point-of-care assay: facilitating a more rapid time to diagnosis. J Infect Dis 2020; 221:2043–9. 31993636 10.1093/infdis/jiaa037

[9] Feld JJ , Lettner B, Mason K, et al Rapid hepatitis C point-of-care RNA testing and treatment at an integrated supervised consumption site in Toronto, Canada. Hepatology 2019; 70:973A–4A. 10.1016/j.lana.2023.100490 PMC10300568 37388709

[10] MacIsaac M , Whitton B, Anderson J, et al Rapid point of care HCV testing allows high throughout HCV screening and rapid treatment uptake among PWID attending a medically supervised injecting room. International Network on Health and Hepatitis in Substance Users. Sydney, Australia. October 13–15. 2021

[11] Freiman JM , Tran TM, Schumacher SG, et al Hepatitis C core antigen testing for diagnosis of hepatitis C virus infection: a systematic review and meta-analysis. Ann Intern Med 2016; 165:345–55. 27322622 10.7326/M16-0065 PMC5345254

[12] Freiman JM , Wang J, Easterbrook PJ, et al. Deriving the optimal limit of detection for an HCV point-of-care test for viraemic infection: analysis of a global dataset. J Hepatol 2019; 71:62–70. 30797050 10.1016/j.jhep.2019.02.011 PMC7014921

[13] van Tilborg M , Al Marzooqi SH, Wong WWL, et al HCV Core antigen as an alternative to HCV RNA testing in the era of direct-acting antivirals: retrospective screening and diagnostic cohort studies. Lancet Gastroenterol Hepatol 2018; 3:856–64. 30274834 10.1016/S2468-1253(18)30271-1

[14] Sun HY , Liu WD, Wang CW, et al Performance of hepatitis C virus (HCV) core antigen assay in the diagnosis of recently acquired HCV infection among high-risk populations. Microbiol Spectr 2022; 10:e0034522. 35579445 10.1128/spectrum.00345-22 PMC9241744

[15] Nguyen LT , Dunford L, Freitas I, et al Hepatitis C virus core mutations associated with false-negative serological results for genotype 3a core antigen. J Clin Microbiol 2015; 53:2697–700. 25994168 10.1128/JCM.01062-15 PMC4508445

[16] European Association for the Study of the Liver. Clinical Practice Guidelines Panel: Chair; EASL Governing Board representative; Panel members. EASL recommendations on treatment of hepatitis C: final update of the series. J Hepatol 2020; 73:1170–218. 32956768 10.1016/j.jhep.2020.08.018

[17] Adee M , Zhong H, Reipold EI, Zhuo Y, Shilton S, Chhatwal J. Cost-effectiveness of a core antigen-based rapid diagnostic test for hepatitis C. Value Health 2022; 25:1107–15. 35272954 10.1016/j.jval.2022.01.004

[18] Dupont SC , Fluker SA, Quairoli KM, et al Improved hepatitis C cure cascade outcomes among urban baby boomers in the direct-acting antiviral era. Public Health Rep 2020; 135:107–13. 31756116 10.1177/0033354919888228 PMC7119255

[19] Bartlett SR , Wong S, Yu A, et al The impact of current opioid agonist therapy on hepatitis C virus treatment initiation among people who use drugs from the direct-acting antiviral (DAA) era: a population-based study. Clin Infect Dis 2022; 74:575–83. 34125883 10.1093/cid/ciab546 PMC8886915

[20] Kronfli N , Linthwaite B, Kouyoumdjian F, et al Interventions to increase testing, linkage to care and treatment of hepatitis C virus (HCV) infection among people in prisons: a systematic review. Int J Drug Policy 2018; 57:95–103. 29715590 10.1016/j.drugpo.2018.04.003

[21] Biondi MJ , van Tilborg M, Smookler D, et al Hepatitis C core-antigen testing from dried blood spots. Viruses 2019; 11:830. 31489933 10.3390/v11090830 PMC6784259

[22] Smookler D , Vanderhoff A, Biondi MJ, et al Reducing read time of point-of-care test does not affect detection of hepatitis C virus and reduces need for reflex RNA. Clin Gastroenterol Hepatol 2020; 19:1451–8.e4 32763480 10.1016/j.cgh.2020.07.058

[23] Vanderhoff A , Smookler D, Biondi MJ, et al Leveraging corona virus disease 2019 vaccination to promote hepatitis C screening. Hepatol Commun 2023; 7:e2101. 36329631 10.1002/hep4.2101 PMC9827963

[24] Adee M , Zhuo Y, Zhong H, et al Assessing cost-effectiveness of hepatitis C testing pathways in Georgia using the Hep C testing calculator. Sci Rep 2021; 11:21382. 34725356 10.1038/s41598-021-00362-y PMC8560949

[25] Zhang Y , Huang Z, Zhu J, Li C, Fang Z, Chen K. An updated review of SARS-CoV-2 detection methods in the context of a novel coronavirus pandemic. Bioeng Transl Med 2022; 8:e10356. 35942232 10.1002/btm2.10356 PMC9349698

[26] Yip CC , Sridhar S, Chan WM, et al Development and validation of a novel COVID-19 nsp8 one-tube RT-LAMP-CRISPR assay for SARS-CoV-2 diagnosis. Microbiol Spectr 2022; 10:e0196222. 36445095 10.1128/spectrum.01962-22 PMC9769742

